# Quantitative Live-cell Reporter Assay for Noncanonical Wnt Activity

**DOI:** 10.21769/BioProtoc.2762

**Published:** 2018-03-20

**Authors:** Edith P. Karuna, Michael W. Susman, Hsin-Yi Henry Ho

**Affiliations:** 1 Department of Cell Biology and Human Anatomy University of California, Davis School of Medicine, Davis, California, USA; 2 Department of Neurobiology, Harvard Medical School, Boston, Massachusetts, USA

**Keywords:** Noncanonical Wnt reporter, Wnt5a signaling, Kif26b, Regulated degradation, Flow cytometry

## Abstract

Noncanonical Wnt signaling functions independently of the β-catenin pathway to control diverse developmental processes, and dysfunction of the pathway contributes to a number of human pathological conditions, including birth defects and metastatic cancer. Progress in the field, however, has been hampered by the scarcity of functional assays for measuring noncanonical Wnt signaling activity. We recently described the Wnt5a-Ror-Kif26b (WRK) reporter assay, which directly monitors a post-transcriptional regulatory event in noncanonical Wnt signaling. In this protocol, we describe the generation of the stable GFP-Kif26b reporter cell line and a quantitative reporter assay for detecting and measuring Wnt5a signaling activities in live cells via flow cytometry.

## Background

Historically, transcriptional reporter assays have facilitated the delineation of major signaling pathways. In particular, β-catenin-dependent luciferase- or GFP-based transcriptional reporters have been instrumental in elucidating the molecular mechanisms of the canonical Wnt/β-catenin pathway (Korinek *et al*., 1997; Fuerer and Nusse, 2010). Although a number of noncanonical Wnt signaling reporters based on JNK-dependent transcription have been described, it remains unclear whether these transcriptional responses are primary or secondary to noncanonical Wnt signaling (Veeman *et al*., 2003, Nishita *et al*., 2010, Ohkawara and Niehrs, 2011). Also, a reporter for real-time detection of non-transcriptional Wnt5a-Ror signaling events has not been available. The Wnt5a-Ror-Kif26b (WRK) reporter assay, which directly monitors a non-transcriptional Wnt5a-Ror signaling event, adds to the current repertoire of molecular tools for studying noncanonical Wnt signaling (Ho *et al*., 2012; Susman *et al*., 2017).

As described in our recent publication, Wnt5a-Ror signaling modulates the steady-state protein level of the kinesin superfamily member Kif26b by inducing its ubiquitin- and proteasome-dependent degradation (Susman *et al*., 2017). This reporter assay enables further identification and mechanism-based analysis of other Wnt5a-Ror signaling components, most of which remain unknown or relatively unexplored. In addition, the WRK assay may also facilitate the screening of pharmacological agents in Wnt5a-Ror related diseases such as certain cancers and developmental disorders.

This protocol describes the generation of the stable GFP-Kif26b reporter cell line and a quantitative method of detecting Wnt5a signaling levels in live GFP-Kif26b reporter cells via flow cytometry.

## Materials and Reagents

1. Pipette tips (USA Scientific, catalog numbers: 1122-1832, 1120-8812, 1123-1812, 1121-3812)
2. 10-cm tissue culture dish (Corning, Falcon^®^, catalog number: 353003)
3. 1.5 ml microcentrifuge tubes (Denville Scientific, catalog number: C2170)
  *Note:* Autoclave before use.
4. 6-well plate
5. 48-well tissue culture plate (Corning, Costar^®^, catalog number: 3548)
6. 5 ml round-bottom tubes with 35 μm cell strainer snap cap (Corning, Falcon^®^, catalog number: 352235)
7. NIH/3T3 Flp-In cells (Thermo Fisher Scientific, Invitrogen™, catalog number: R76107)
8. pCAG-GFP (available upon request), or any GFP plasmid suitable for mammalian expression
9. pEF5-FRT-GFP-Kif26b (Addgene, catalog number: 102862) reporter construct
10. pOG44 Flp-Recombinase expression vector (Thermo Fisher Scientific, Invitrogen™, catalog number: V600520)
11. Recombinant Wnt5a (R&D Systems, catalog number: 654-WN-010)
12. Genjet *In Vitro* Transfection Reagent for NIH/3T3 cells (SignaGen Laboratories, catalog number: SL100488, 3T3)
13. Hygromycin B (50 mg/ml solution) (Corning, Mediatech, catalog number: 30-240-CR)
14. Poly-D-lysine (Sigma-Aldrich, catalog number: P6407-10X5MG)
15. Wnt-C59 (Cellagen Technology, catalog number: C7641-2s)
16. Trypsin EDTA (Corning, Mediatech, catalog number: 25-052-CI)
17. Dulbecco's modified Eagle's medium (DMEM) (Corning, Mediatech, catalog number: 15-017-CV)
18. Fetal bovine serum (FBS) (Thermo Fisher Scientific, Gibco™, catalog number: 16000069)
  Note: The FBS is used directly without heat-inactivation.
19. Glutamine (100× solution, 200 mM) (Corning, Mediatech, catalog number: 25-005-CI)
20. Penicillin-streptomycin (100× solution, 100 IU/ml) (Corning, Mediatech, catalog number: 30-002-CI)
21. Bovine serum albumin (BSA) (Fisher Scientific, catalog number: BP1600-1)
22. CHAPS detergent (Thermo Fisher Scientific, Thermo Scientific™, catalog number: 28300)
23. Phosphate buffered saline (PBS) (GE Healthcare, catalog number: SH30256.01)
24. Growth media (see Recipes)
25. Wnt control buffer (see Recipes)
26. Cell resuspension buffer for flow cytometry (see Recipes)

## Equipment

1. Pipetters (*e.g*., Eppendorf, model: Research^^®^^ plus)
2. 37 °C, 5% CO_2_ incubator (*e.g*., Heracell by Thermo Fisher Scientific)
3. Centrifuge with cooling capabilities (*e.g*., Thermo Fisher Scientific, Thermo Scientific™, model: Sorvall™ Legend™ Micro 21R)
4. Fluorescent microscope with 488 nm light source (*e.g.*, Thermo Fisher Scientific, model: EVOS^^®^^)
5. Flow cytometer with 488 nm laser (*e.g*., BD, model: FACScan)

## Software

1. FlowJo software (FlowJo, LLC; https://www.flowjo.com/)

## Procedure

**Figure 1:**
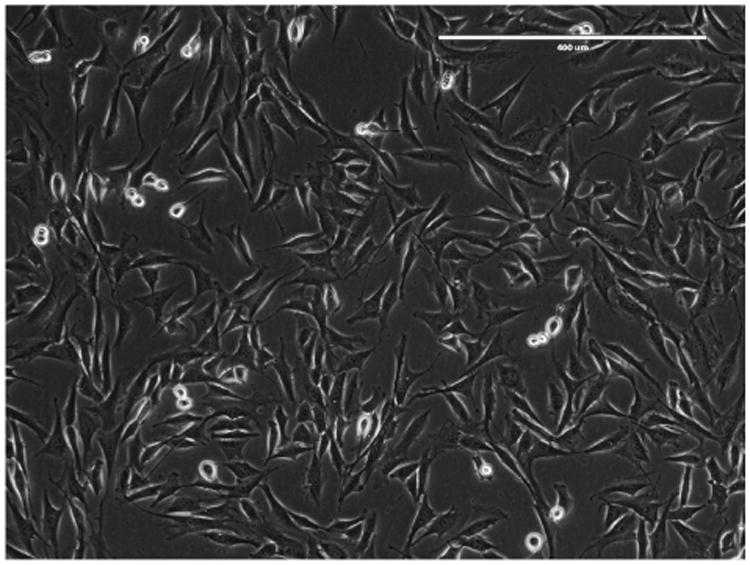
Confluency (80%) at the time of transfection Phase contrast, 10× magnification. Scale bar represents 400 μm.

**Figure 2:**
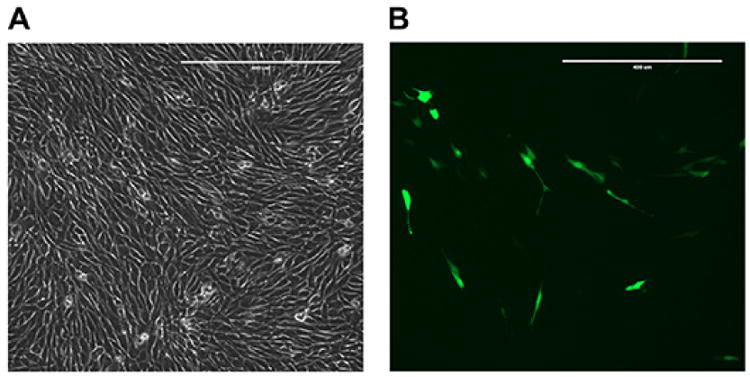
GFP control plate 12-18 h after transfection A. Phase contrast channel, 10× magnification. Scale bar represents 400 μm. B. GFP channel, 10× magnification. Scale bar represents 400 μm.

**Figure 3:**
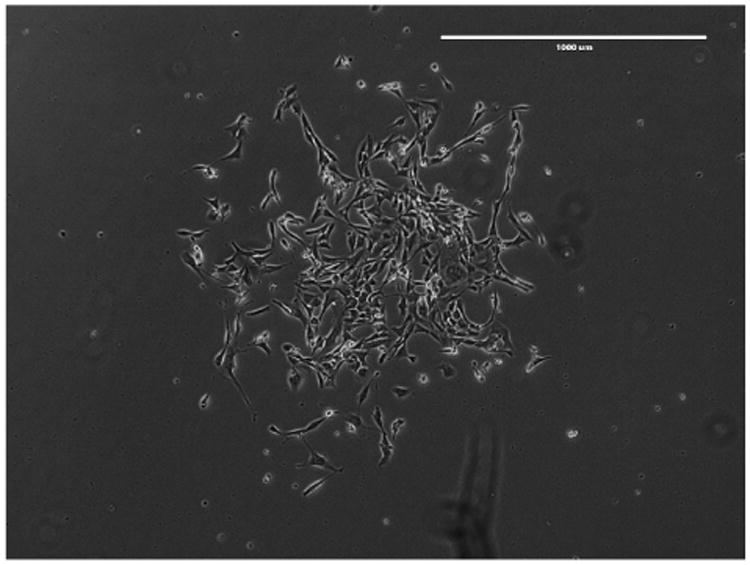
A representative colony at 7 days post-hygromycin B selection Phase contrast, 4× magnification. Scale bar represents 1,000 μm.

- Generation of stable reporter cell lines using the Flp-In NIH/3T3 cell line
  1. Cell plating for transfection
    Seed cells at 1.62 M cells/plate in a 10-cm plate in 10 ml of growth media. Culture the cells at 37 °C until they reach 80% confluency (about 18-24 h, Figure 1).
    Note: Prepare 1 plate of cells for each reporter construct, plus 1 additional plate for the pCAG-GFP, which serves as both a negative control for the Flp-In and a reference for transfection efficiency.
  2. Transfection
    - One hour before transfection, remove media from cells and replace with 6 ml fresh growth media.
    - Dilute DNA: In a 1.5 ml microcentrifuge tube, add 1.35 μg pEF5-FRT-GFP-Kif26b and 12.15 μg pOG44 to 675 μl of serum-free media (plain DMEM). In parallel, for the GFP control plate, prepare a tube of 675 μl serum-free media with 13.5 μg of pCAG-GFP but no pOG44. Mix well by pipetting.
      Note: Total mass of transfected DNA is 13.5 μg. Transfect with a 1:10 molar ratio of reporter plasmid to flp recombinase; adjust masses according to the size of the plasmid.
    - Dilute the GenJet transfection reagent: for each plate, prepare a separate 1.5 ml microcentrifuge tube of 40.5 μl GenJet transfection reagent in 675 μl of serum-free media (plain DMEM). Mix well by pipetting.
    - Add each tube of diluted GenJet solution all at once to each respective DNA solution.
      Note: The GenJet solution must be added to the DNA solution, not the reverse. Vortex gently for 4 sec to mix.
    - Incubate the transfection mixes for 15 min at room temperature. Do not let the incubation proceed for more than 20 min.
    - Add the transfection mixes drop-wise to their respective plates of cells.
    - Gently rock the plates to mix well and return the plates to the incubator.
    - After 12-18 h, check transfection efficiency by visualizing the GFP control plate under a fluorescent microscope (Figure 2). Remove transfection media and replace with 10 ml of growth media.
  3. Antibiotic selection
    - Two days after transfection, split each 10-cm plate into 4 × 10-cm plates in growth media to avoid overcrowding cells during selection (do not use selection antibiotics during the split).
    - After cells adhere to the plate, remove media and replace with fresh growth media containing 200 μg/ml hygromycin B. Replace with fresh hygromycin media every 3-4 days. Selection should take about 7-10 days. Between 6-20 colonies per plate is typically expected (Figure 3).
      Note: A kill curve was conducted to determine that 200 μg/ml hygromycin B is optimal for NIH/3T3 Flp-In cells. The optimal selection concentration may vary slightly depending on the source of hygromycin B and cell lines.
    - Cells may be pooled from 1 or 2 10-cm plates into a single well of a 6-well plate and passaged in growth media without selection antibiotics.
      Note: This step is only performed for the reporter constructs. The GFP control plate, which should yield no colonies, is discarded.
- Wnt5a stimulation assay
  Experimental design: For a basic Wnt5a stimulation, include one condition for stimulation (+Wnt5a, where Wnt5a-containing media is added) and one condition for control (-Wnt5a, where control buffer-containing media is added) for each reporter cell line. The experiment setup will vary depending on your application of the assay; see Data analysis section for details on other types of stimulations.
  1. Seed reporter cells at 0.09 million/well in the poly-D-lysine-coated 48-well plate in 400 μl growth media per well. Cells should be about 90% confluent.
    Notes:
    - Plate coating is done by adding 200 μl of a poly-D-lysine solution (0.1 mg/ml in water; sterile filtered) to each well of a 48-well plate, incubating at room temperature for 15 min, removing the poly-D-lysine solution, and washing the wells with 400 μl of water three times. Air dry the plate completely (with the lid removed) before plating cells. Coated plates can also be stored at room temperature for future use.
    - For quantification, we typically plate cells in triplicate wells for each experimental condition.
  2. The next day, gently remove media and replace with 400 μl growth media containing 10 nM Wnt-C59. Wnt-C59 inhibits the processing and secretion of endogenous Wnts. Allow cells to reach 100% confluency in Wnt-C59-containing media (generally one day). Cells should be as confluent as possible on the day of Wnt5a stimulation.
    Note: If the monolayer of cells retract or peel off, repeat cell plating. Retracted cells do not signal well.
  3. To stimulate cells with Wnt5a, gently remove media and replace with media containing 10 nM Wnt-C59 and the respective concentration of Wnt5a. For mock stimulation, use media containing Wnt-C59 and Wnt control buffer. If other drugs are used in conjunction with Wnt5a, pretreatment of the drug (typically for 1 h) may be necessary before addition of Wnt5a- and drug-containing media. Avoid disturbing the cell monolayer during media change.
  4. Incubate cells with Wnt5a at 37 °C for 6 h.
  5. To harvest cells for flow cytometry analysis, dissociate the cells with 100 μl trypsin per well at 37 °C for 3-5 min. Neutralize the trypsin with 500 μl of growth media and transfer the cell suspensions to 1.5 ml microcentrifuge tubes.
  6. Centrifuge cells at 12,000 *× g* at 4 °C for 3 min to pellet the cells.
  7. Remove the supernatant from each sample. Avoid disturbing the pellet.
  8. Resuspend the pellets at room temperature in 100-150 μl flow cytometer buffer. Mix by pipetting until the sample is homogenously resuspended and strain the cell suspension into a round-bottom tube through the strainer cap.
  9. Analyze the cells using a flow cytometer. We routinely use the Becton Dickinson FACScan and analyze 30,000 cells per sample.
  10. Analyze data files in software (*e.g*., FlowJo). See next section for details.

## Data analysis

**Figure 4:**
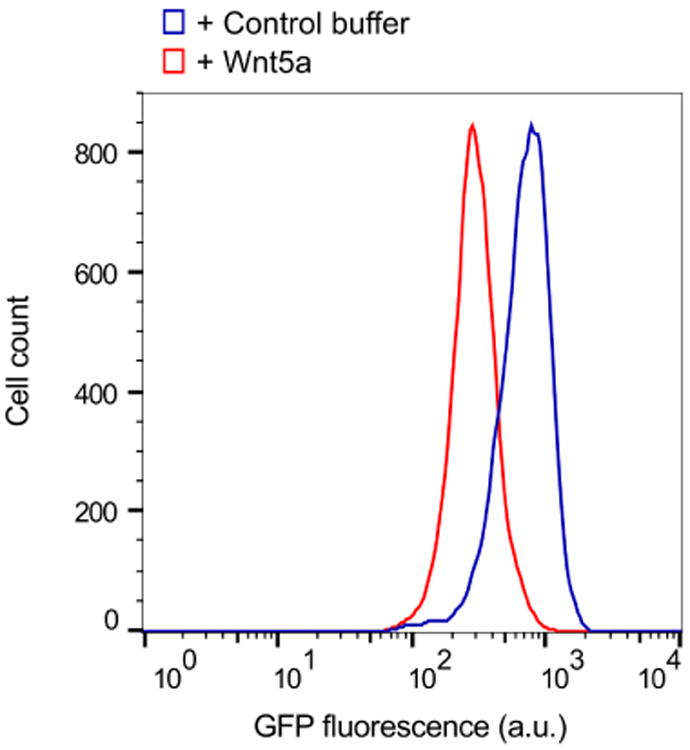
Basic analysis using the WRK reporter assay Overlaid histograms from one set of samples showing the downregulation of GFP-Kif26b fluorescence in the WRK reporter cell line after Wnt5a stimulation (0.2 μg/ml Wnt5a) for 6 h.

**Figure 5:**
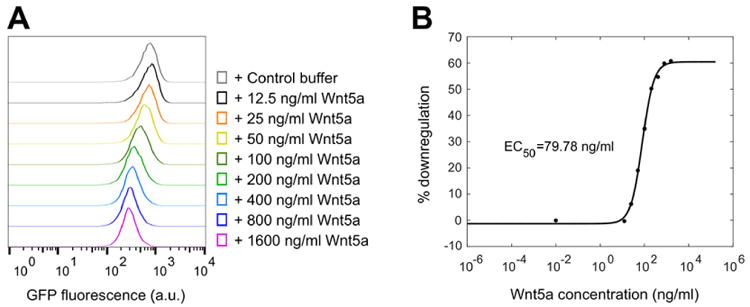
Example of a dose-response analysis using the WRK reporter assay Raw histograms (A) and the resulting dose-response curve (B) showing GFP-Kif26b downregulation as a function of Wnt5a concentration in the WRK reporter assay.

**Figure 6:**
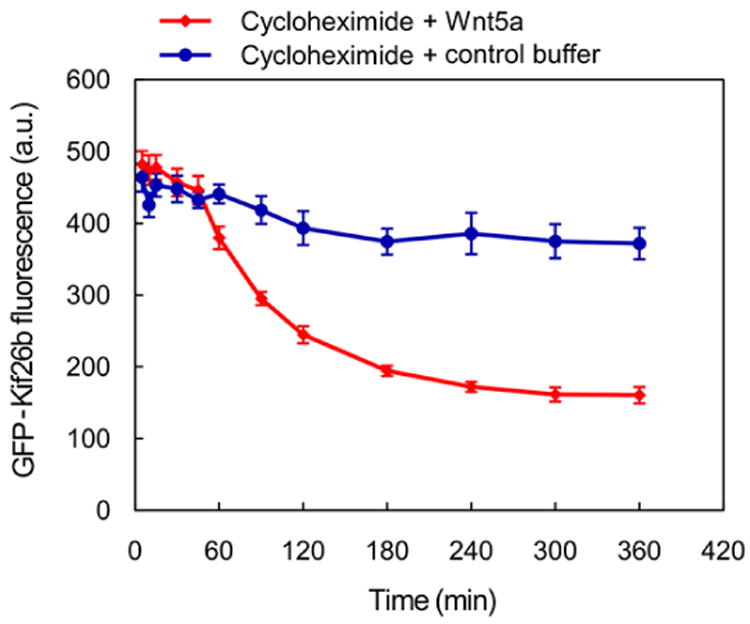
Example of a time course experiment using the WRK reporter assay The kinetics of GFP-Kif26b turnover in the absence or presence of Wnt5a stimulation, as measured in the WRK reporter assay. Cycloheximide was used to block new protein synthesis in the reporter cells.

**Figure 7:**
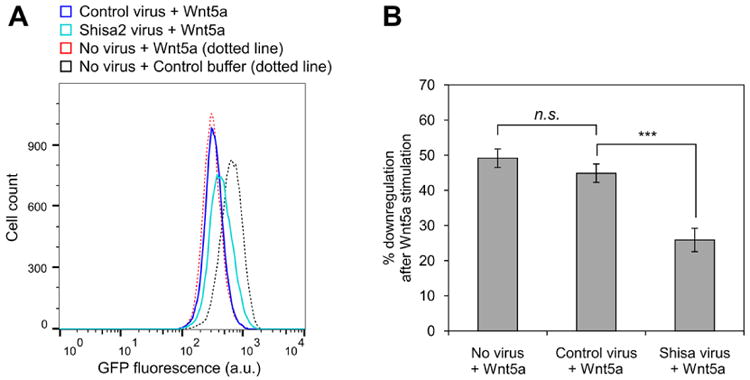
Example of a pathway analysis experiment using the WRK reporter assay Partial blocking of Wnt5a-induced reporter activity after ectopic Shisa2 expression via lentiviral transduction. A. Representative overlaid histograms show the effect of ectopic Shisa2 expression on Wnt5a-induced downregulation of GFP-Kif26b in the WRK reporter line. Shisa2 is an antagonist of the Frizzled family of Wnt receptor (Yamamoto *et al.*, 2005). The effect of Wnt5a or control buffer treatment on the WRK reporter line is included as a reference. B. Quantification of the results shown in panel (A). *T*-tests were performed for the following comparisons: Control virus vs. no virus, *P* = 0.0957 (not significant); control virus vs. Shisa2 virus, *P* < 0.001 (significant).

1. For general data analysis, gate the live cell population via side scatter and forward scatter parameters in the flow cytometry software to exclude dead cells. The wild-type NIH/3T3 Flp-In parent cell line (*i.e.*, untransfected) is used as a reference for autofluorescence; however, we do not typically gate the cell population based on the GFP signal to ensure that the entire live cell population is included in the reporter analysis. Generate a raw histogram of GFP fluorescence vs. cell count for the live gated population. Overlay the histograms from each sample to be compared to obtain the difference in median fluorescence between each sample population (Figure 4). This difference in medians is expressed as a percentage: [(Control med an - stimulated median)/control median] × 100 (labeled as ‘% downregulation’ in Figure 5B). Multiple histograms may be overlaid for comparison or reference (Figure 5A, Figure 7A).
2. For a dose-response analysis, we analyze a minimum of six samples with varying concentrations of the Wnt5a ligand or small molecule inhibitors, including a 0 dose point. The medians may be plotted against the concentrations to generate the dose-response curve (Figure 5B). For inhibitors, we typically vary the drug concentration in the presence of a fixed concentration of Wnt5a to determine the dose-response relationship.
3. For a time course experiment, such as the Kif26b stability analysis shown in Figure 6, we stimulate samples with Wnt5a at regular time intervals until the end of the experiment, when all samples are harvested at once. The medians are plotted against the duration of stimulation.
4. For statistical analysis during quantification, we use a minimum of three biological replicates (cells plated and treated with Wnt5a and/or inhibitors in concurrent cultures). To assess the difference between two sets of data, we perform a two-tailed, unpaired Student's *t*-test (Figure 7B). We include error bars for each set of replicates representing the standard error of the mean, which we generate by calculating the standard deviation of the medians of the replicates and dividing that number by the square root of *N*, where *N* is the number of replicates (Figure 6, Figure 7B).

## Notes

Wnt5a signaling as detected by this assay appears to be highly sensitive to cell density. Signaling activity occurs best when cells are as confluent as possible, and activity decreases drastically when cells are less than 100% confluent. Some optimization may be required to determine the most optimal plating conditions for specific cell types and applications.

## Recipes

1. Growth medium
  DMEM supplemented with:
  10% FBS
  1× glutamine (2 mM)
  1× penicillin-streptomycin (1 IU/ml)
2. Wnt control buffer
  1× PBS supplemented with:
  0.1% bovine serum albumin
  0.5% (w/v) CHAPS
3. Cell resuspension buffer for flow cytometry
  1× PBS supplemented with 0.5% FBS
